# The Long Fox Memorial Lecture: The Influence of Food on the Production and Prevention of Disease

**Published:** 1930

**Authors:** J. A. Nixon

**Affiliations:** Professor of Medicine in the University of Bristol, Physician to the Bristol Royal Infirmary, Consulting Physician to Southmead Hospital


### The Bristol Medico-Chirurgical Journal

" *Scire est nescire, nisi id me*
*Scire alius sciret.*"

WINTER, 1930.

### THE LONG FOX MEMORIAL LECTURE:

DELIVERED IN THE PHYSIOLOGICAL LECTURE THEATRE OF THE UNIVERSITY ON OCTOBER 28TH 1930.

THOMAS LOVEDAY, LL.D., *Vice-Chancellor of the University of Bristol, in the Chair.*

BY

J. A. Nixon, C.M.G., M.D., F.R.C.P.,
*Professor of Medicine in the University of Bristol,*
*Physician to the Bristol Royal Infirmary,*
*Consulting Physician to Southmead Hospital,*

ON

### THE INFLUENCE OF FOOD ON THE PRODUCTION AND PREVENTION OF DISEASE.

---

Mr. Vice-Chancellor, Ladies and Gentlemen, to-night we are assembled to honour the memory of Edward Long Fox, the third of that name and line who held the office of Physician at the Bristol Royal Infirmary. His writings and the testimony of those who knew him bear witness to his great powers of

T

keen, patient and honest observation which place him amongst the most scientific doctors of his time, and beyond this we know that he possessed those cardinal virtues of a great physician, a soundness of understanding and an ability to apply his knowledge instantly and aright in practice. His character and work were justly held in high esteem during his lifetime, and this lecture was established in order that we might acknowledge year by year the debt we owe to his labours, his teaching and his example.

The subject I have chosen for my address is "The Influence of Food on the Production and Prevention of Disease." Sir George Newman, in his introduction to a Ministry of Health report on *Diet and Cancer*,[1] remarked that it is one of the commonest of human prejudices to assume a causal relation between various habits and customs practised by men and the diseases from which they suffer. Among such customs those relating to dietary take a prominent place, and it would seem obvious that deviations from the normal working of the body should be related to the nature and quantity of the food consumed.

I propose to consider what evidence there is that the nature and quantity of the food we consume hurts or helps the normal working of the body. The accidental contamination of food by extraneous poisons, harmful parasites or micro-organisms will not be discussed. The facts are for the most part well established and the effects fully proved. I do not intend to deal with such foods nor those articles which contain endogenous poisons that may be taken as food in error, like poisonous fishes or fungi.

The term ptomaine poisoning has been used popularly of all sorts of food poisoning, but true ptomaine poisoning is very rare, and in fact is scarcely likely to be met with except from the eating of poisonous mussels which contain mytilotoxin, one of the most poisonous of known ptomaines. Putting food poisons of this class aside, there is still a vast field for inquiry into the effect of food on the human body in relation to the maladies from which men suffer. There has been a considerable increase of knowledge regarding the effect of dietaries on nutrition in general and in particular morbid conditions. The spread of a smattering of knowledge combined with prejudice, speculation and credulity has given rise to an amazing amount of food faddism and inspired hosts of food cranks.

Scientific data are now beginning to emerge from a forest of phantasy, which show that health and sickness are in certain directions intimately related to composite dietaries and individual foodstuffs.

*Food Allergy.*

Urticaria, or nettle-rash, has long been recognized to be due to some peculiarity in the individual quite as much as to inherent properties in the article of food. The term "allergy" was coined to describe this individual peculiarity, and is defined as a condition of unusual or exaggerated susceptibility to a substance which is harmless in similar amounts for the majority of members of the same species. Anaphylaxis, it may be recalled, is a phenomenon produced in laboratory animals by injection of a foreign protein which so sensitizes the animal that a second injection of the same protein produces toxic symptoms. This phenomenon is very difficult to produce in man. Allergy, however, is frequently met with. The sensitiveness is not confined to proteins, and it may manifest itself after ingestion of the offending material or after injection or inhalation. As a response to the ingestion of food the results may be most diverse. Serum sickness is probably familiar to us all; we are well acquainted with the rashes that may occur, mainly of an urticarial or erythematous character, sudden collapse, faintness, even death, vomiting, diarrhœas, abdominal pains, joint pains and synovial effusion, œdema either localized or general. Precisely the same variety of symptoms may follow the eating of certain foods in susceptible individuals, yet the foods may be quite harmless in similar quantities to the majority of people. It is the exception to see allergic phenomena set up in human beings by previous introduction of the offending material after the manner of laboratory anaphylaxis; the response in a susceptible person occurs at the first introduction of the food. Yet it is not always so; excess in a healthy and previously insusceptible adult may produce sensitiveness to many food stuffs. Professor Walker Hall will, I know, pardon a personal reference to his experience. For he was able by taking an incredible number of eggs in the day at last to sensitize himself so that it took him some years before he could touch the smallest amount of egg in any form without the most intense urticaria. So, too, I have seen an adult woman treated by strict dieting on glucose and water for *B. coli* infection of the kidneys develop an amazing urticarial response to glucose. Small children may become sensitized to eggs, apples, bananas and the like by too frequent repetition, so that they develop "heat spots" whenever they eat these articles, and yet when given the same foods at longer intervals no spots appeared. It is, however, more usual for these idiosyncrasies to manifest themselves in human beings at the first ingestion of particular foods, just as the severest outbursts of serum sickness occur in man at the first injection.

Food allergy not only takes the form of skin eruptions; asthma, migraine and epilepsy have been traced to this cause. The precise mode of production of the allergic response is far from being explained, though Sir T. Lewis's researches [2] indicate that by the circulation of some intermediate substance which is not itself histamine the tissue cells in certain areas produce histamine and the histamine evokes a local œdema.

According to one theory, the attack of acute gout is an allergic response to particular articles of food; but whilst the typical response in other allergies is a local œdema, in gout the local response is a deposit of sodium biurate. At the same time, one must not be too sweeping in saying that the allergic response is always œdema, for in Henoch's purpura hemorrhages occur, in cyclical vomiting of children there is no evidence of local œdema, and in asthma the respiratory difficulty is probably due to spasm of the bronchial muscle without local œdema. Although this group of diseases may in some instances be checked by discovering a food allergy through the well-known cutaneous tests, our hopes of relieving every case by this means have all too often proved vain.

*Diet and Cancer.*

The belief that there is a causal connection between diet and cancer dies hard, especially in the propaganda of various would-be "food reformers." Meat eating has been proclaimed far and wide as the one sure cancer producer: the statement that primitive non-meat eating races are free from the scourge of cancer has been repeated until many people accept the statement as a scientific fact. In recent years a few careful observations have been made which show how unfounded this belief is. Naumann[3] reports that in Jeremie (a remote district in the island of Haiti) he has investigated a population of 10,000 inhabitants living on fresh fruit and vegetables with little meat or fat, and these primitive people, on a vitamin rich diet, do not show low cancer figures. Monckton Copeman and Major Greenwood,[1] at the instance of the Ministry of Health, conducted an inquiry into the question whether the members of certain religious communities who abstain from flesh food showed a low incidence of cancer. Their report states that fatal cancer occurs in such populations, and that their analysis lends no real support to the contention that the relative incidence of cancer amongst them is low. The statement that cancer is unknown amongst the primitive and vegetarian tribes of Southern India has been proved to be incorrect.[4] Finally, Murray, in the *Report of the Imperial Cancer Research Fund for* 1928,[5] stated categorically that there is no reliable evidence—experimental, statistical or clinical—which would indicate a causal correlation between cancer and the absence or presence or the excess of any particular dietetic constituent.

*Diet and Growth.*

The connection between diet and growth has been appreciated no doubt ever since man appeared upon this planet. Mothers throughout the ages have realized that the growth of their infants both in stature and in wisdom was directly related to their food. With an absolute insufficiency of food growth in every direction is retarded or even arrested. The knowledge that the lack of particular constituents in the diet could interfere with normal growth is, however, comparatively new. Yet it was thought even within our own times that so long as the diet contained a certain quantity of protein for tissue formation and adequate "carbonaceous" food, as it used to be called, to furnish the calories for energy normal growth would result. At last it began to be noticed that even though the diet contained protein, fat and carbohydrate in amounts and proportions that were chemically correct the results sometimes fell far below expectation. It was only at the beginning of the twentieth century that Rohmann[6] found that if purified materials such as casein, egg-albumen, vitellin, potato starch, wheat starch and oleomargarine, together with the proper salts, were mixed and given to mice, their offspring were difficult to rear and that no living young could be obtained from them. In 1905 Pekelharing,[7] experimenting also with mice, pointed out that there was a still unknown substance in milk which even in very small quantities was of paramount importance to nutrition. Gowland Hopkins[8] in 1906 wrote: "No animal can live on a mixture of pure protein, fat and carbohydrate, and even when the necessary inorganic material is carefully supplied the animal still cannot flourish." He mentioned that in such diseases as rickets and scurvy it had long been known that there was some obscure dietetic factor at work. Out of these observations has arisen all our knowledge of those accessory food factors which are now called vitamins. For proper growth it is requisite that the diet shall contain sufficient energy in the form of calories to maintain the organism and, in addition, sufficient vitamins, salts and protein, and further that the protein must be of good biological value. By biological value is meant the value of food proteins for forming or sparing body protein. On the whole, meat, egg, fish and milk proteins are greatly superior to vegetable proteins in this respect.

The reason for differences in biological value of proteins lies in their amino-acid content. One of the poorest known is that obtained from maize, and it lacks tryptophan, lysin and glycin. The addition of tryptophan and lysin to a diet in which the predominant protein was zein (the chief protein of maize) caused normal growth in mice, whilst without these additions the animals declined and died.[9] Lysin alone does not prevent their decline, and the addition of tryptophan by itself did no more than maintain their weight without normal increase. Histidin also is essential to growth.

The protein of whey is one of the best materials for growth, this may be due to the larger amount of cystin it contains. The addition of cystin to biologically poor proteins will improve growth. Similarly, wheat protein has a relatively high value on account of the glutenin it contains. At any rate, enough is now known to enable us to select foods containing growth-producing proteins and to avoid those of poor biological value. Something more than protein is needed for growth, however, there must be certain salts and there must be sufficient vitamins even though the amounts of the latter may be minute. The principal vitamin necessary for normal growth is A, the chief sources of which are animal fats, green leaves, milk and the internal glandular organs, the liver and kidney. Cod liver oil is the richest source of A. It is thought that the lipochrome carotene, the yellow colouring of carrots, is allied to, if not identical with, vitamin A. It is essential for the growth of the child that the diet shall contain this and other vitamins, together with sufficient protein and sufficient calories, not merely to maintain the metabolism at rest, but in many cases to allow of a surplus to be fired off in exercise and in crying. Roughly the caloric needs of a child during the first year of life are 80 calories per kilogram of body weight and the protein need is 3·5 grams per kilo. A general arrest of growth may be caused by insufficiency of the building materials and of the energy to build them together. There are, however, other defects of growth which may rightly be termed disease that depend upon building the body imperfectly without actual arrest of growth.

*Diet and Rickets.*[10]

Rickets was one of the first diseases to be distinctly attributed to feeding defects. I need not go through the whole description of the condition. The outstanding feature is failure of calcification of the bones, which is associated with a deficiency of phosphorus in the blood and a normal calcium content. The remedy was known long before the cause of the condition. Cod liver oil was a specific remedy before vitamins had been heard of. Now we recognize that the absence of vitamin D is the principal factor in causing rickets, together with faulty proportions of calcium and phosphorus in the food.

The bone defects, tetany and laryngismus stridulus, which are associated with rickets can all be cured by the addition of D to the diet. D may be given in the form of cod liver oil, irradiated ergosterol (viosterol) or as ultra-violet radiation, even better, real sunshine. In later life a lack of vitamin D may produce osteomalacia and mollities ossium.[11] Vitamin D is, as Mrs. Mellanby[12] has demonstrated, a most powerful agent in promoting the calcification of teeth and in preventing dental caries in children. Cereals, and oatmeal especially, inhibit perfect calcification when D

is deficient in the diet. Vitamin D is supplied to the breast-fed infant in the mother's milk, provided the mother herself is receiving an adequate allowance of it. In many instances rickets has begun in intra-uterine life, and prevention should start by the treatment of the pregnant mother.

*Diet and Pregnancy.*

Thus we must look to the health of the mother as the first step towards the prevention of rickets. How far can we influence the woman either in anticipation of pregnancy or during it? I cannot do better than quote Caffey[13] on this subject: "A mother during pregnancy and lactation should have a diet abundant in vitamins A, B, C and D, whilst her environment should provide adequate exposure to sunlight or ultra-violet radiation." He advocates much more attention to these requirements during the ante-natal period as well as during lactation.

It is a mistake to suppose that during pregnancy attention need only be paid to the vitamin content of the mother's diet. There are other conditions than avitaminosis which are inseparably connected with diet. The toxæmias of pregnancy may in some instances be produced by faulty dietary, and in many cases if the danger is recognized early enough an adjustment of diet will avert disaster. This is not the place or time to discuss the whole etiology of albuminurias of pregnancy or of eclampsia. Some of them are undoubtedly due to a pre-existing renal defect. Others appear to depend upon hepatic insufficiency. My experience leads me to think that when permanent renal changes can reasonably be excluded from the diagnosis the occurrence of eclampsia can largely be controlled by diet. Professor Walter Swayne used to point out how often a woman came into the Infirmary in eclampsia or with threatening of it following directly after a meal containing an unusually large quantity of pork. The custom of urging expectant mothers to consume as much fat as possible may also be responsible for some of the toxic symptoms met with, particularly vomiting late in pregnancy and the appearance of ketones in the urine. Such cases will almost invariably improve if their diet is changed to one in which carbohydrates predominate.

It is now a well-established principle that in order to protect the liver against hepatic poisons it should be enriched with as great a store of glycogen as possible. This is a widely-practised precaution against the toxic effects of the organic arsenic compounds so freely used in V.D. clinics and against the ill-effects occasionally seen after tetra - iodo - phenolphthalein injection.

Protein and fat metabolism seem to go astray early when the liver functions are deranged, whilst carbohydrate metabolism is sustained for some time longer.

Fertility in the mother (and perhaps the father) depends in animals upon a sufficiency of vitamin E. Actually E is so widely distributed through all the foodstuffs in ordinary use that it is probably very rarely that a human being suffers from lack of it, and the causes of infertility must usually be sought elsewhere. Miss Chick and Dr. Dalyell,[11] however, reported from Vienna that during the starvation period at the end of the war amenorrhœa and infertility was common in the female population.

Lactation has been the special study recently of Dr. Hoobler[14] of Detroit. This work is really only just beginning, and there are many old wives' fables to be tested and perhaps removed from the category of truths. So far as Hoobler has gone at present his conclusions as to the factors influencing human milk production are as follows :—

The protein portion of the diet should be maintained at a higher level than at other times. For dairy cattle this proportion has been fixed at one part of protein for five or six of fat and carbohydrate reckoned in terms of calories.

Three thousand calories per day seems the optimum intake for a nursing mother.

All vitamins play a part in milk production, but A, B and E seem to have a greater influence than C and D.

Stripping the breasts after each feeding is the best way of maintaining a milk supply or for increasing a failing one.

Ultra-violet rays, according to some observers, may help in keeping up a milk supply.

Absence of worry, fears and unpleasant environment are important, but not germane to this lecture.

Whilst dealing with the subject of lactation it is worth while to refer to some work of Hoefler,[15] who compared the physical and mental development of breast-fed and artificially-fed children. On the average the artificially-fed were the most susceptible to diseases of childhood. In mental development the lowest class were those who were breast-fed from ten to twenty months. Children breast-fed from four to nine months were superior mentally and physically to all other groups.

*Diet and Alimentary Disease.*

It might appear simple to speak dogmatically of the influence of diet on the alimentary tract; the truth is that in no department has there been more theorizing and less proven fact. An acute gastritis may be set up by partaking of unripe fruit and frankly indigestible substances. Some spices and alcoholic beverages may act as irritant poisons act. I do not intend to include drink in this lecture on food. The plain fact is that the stomach is marvellously tolerant of what is put into it, relying possibly upon its powers of showing its independence of its owner by emptying itself by the act of vomiting whether the owner consents or not. Irregularity of meals and overloading is much more to blame for diseases of the stomach than the actual nature of the food. The stomach can tackle almost any meal if allowed time, or if the food is unsuitable it is rejected. It is singular that among the various factors blamed for the production of gastric and duodenal ulcers the nature of the food hardly comes under serious discussion. When cures are under discussion the case is altered entirely. Yet even here there is a lack of ascertained facts. Riegel,[16] writing in the early days of this century, said: "It is frequently stated that the diet of a case of stomach disease should be easily digestible. It is an easy matter to determine what is easily digestible in a *healthy* subject, but not so simple in *disease* of the stomach." Leube and Penzoldt[16] formulated diet scales according to the length of time various articles remained in the stomach, the former in cases of stomach disease and the latter for healthy stomachs.

All that we know with certainty about peptic ulcers is that in many instances frequent feeding with food found suitable to the individual patient, accompanied by the administration of alkalis, will relieve the suffering and simultaneously the ulcer may heal. The point in such treatment which belongs properly to this lecture is that it is of the utmost importance to see that the diet contains sufficient protein for tissue maintenance and repair, sufficient calories and, most of all, adequate vitamins. I have seen a patient under treatment for gastric ulcer develop xerophthalmia, corneal ulceration and lose her eye because the fat content (that is to say, vitamin A) was deficient.

Our knowledge of the effects of various dietaries upon the intestine, small and large, is also not very sure or extensive. The powers of adaptation appear to be very nearly unlimited as regards the form in which food is taken into the alimentary tract, so long as the proportions of the constituents are correct (and the correct proportions are not inelastic). In parts of West Africa a diet of ripe bananas is sufficient for laborious work,[17] and at the other end of the scale comes the Eskimo, who contrives to live healthily on an all-flesh diet.

The ill-effects of diets which produce diarrhœa are known. Sometimes a severe colitis may be set up. The least harm they do is to pass the bowel contents out so rapidly that nutritious material may escape digestion and assimilation and thus be wasted. This is one of the disadvantages attendant upon the cult of roughage. There is no universal standard, no optimum roughage content which can be applied. Some people feel better when their insides are filled up with indigestible cellulose, others are desperately uncomfortable. Rubner's[18] classical work on war bread in Germany proved that the loss in calories by the fæces is 7 per cent. greater with whole wheat bread than with white, for of the energy content of white bread only 4·5 per cent. appears in the fæces, of whole wheat bread 11·5 per cent. Rubner advocated giving bran to the cattle and eating the beef in order to avoid the waste in the intestinal tract of a human being.

The cow is a poor example to follow in these matters, only 4·5 per cent. of the energy contained in the hay is of actual use in cattle feeding. Similarly and surprisingly, it is found that of vegetable foods the highest wastage of the energy content occurs with strawberries, and the lowest with fine wheat flour. It is an economic crime to preach to the poor that there is a supposed advantage in paying for non-assimilable cellulose padding with their food. As Lusk[19] wrote: "The British people during the war felt that whatever else happened they would return to white bread as soon as they possibly could. Here instinct, supported by science, triumphs once again over ignorance and bigotry."

The ill-effects due to a constipating diet are debatable. The symptoms regarded as characteristic of "intestinal autointoxication" are, in part at least, due to distension and mechanical irritation of the rectum, and may be induced by packing the rectum with masses of cotton wool or barium.[20] Nevertheless, although the explanation frequently advanced of the distressing symptoms of constipation may be incorrect, the fact remains that in many individuals food of a nature to cause constipation gives rise to a definite symptom-complex which is relieved by resorting to a more laxative diet.

*Diet and Blood Diseases.*

Secondary anæmia may be brought about by starvation depending either upon a positive insufficiency of food or upon conditions which interfere with the swallowing or proper digestion of food. In cases such as these provision of adequate food or remedying the defective reception or digestion of food will cure the anæmia. Anæmia is one of the symptoms of rickets, and will disappear with the appropriate treatment of the rickets, always provided that the food has a sufficient iron content, and milk by itself will not cure the anæmia because it does not contain iron.

Chlorosis used to be attributed to "improper feeding." It was common in this country before the war, and has now practically disappeared. There is no good evidence that the dietary of the classes formerly liable to chlorosis has materially changed since the war, so that this disease cannot well be assumed to have a dietetic origin. It went out of fashion when short skirts and pillion riding came in.

Pernicious anæmia is a disease which has provided one of the most interesting dietetic studies. The cure was discovered almost accidentally, and we still know nothing about its causes, beyond the observation that in nearly all cases there is a total absence of HCl from the gastric juice. It is now well known that the administration of liver in the diet will cure the anæmia and protect the patient against relapse so long as the liver diet is continued. Liver does not actually cure the disease, but supplies something which the organism cannot supply without artificial aid. Liver is not the only way of making good the deficiency, an extract of hog's stomach (manufactured under the name of ventriculin) is also effectual; and experiments have shown that if food is first chewed and swallowed by a normal individual, and then after digestion has proceeded a certain time the semi-digested food is withdrawn by a stomach tube and administered to the sufferer from pernicious anæmia, the same improvement occurs as under liver treatment.

Brem, Zeiler and Hammack [21] have recently published a case of duodenal ulcer in which repeated blood transfusions were given for recurrent hemorrhages. Blood from non-fasting donors caused severe febrile reactions and on one occasion hæmoglobinuria, whereas transfusions from fasting donors were not followed by reactions. There were no errors in blood-grouping.

*Diet and Vascular Disease.*

Arterial degeneration, arterio-sclerosis and atheroma have been most confidently attributed to foods and feeding. Everybody is accustomed to hear that the chief cause is meat-eating. The Eskimos live on an almost exclusive meat diet, but Thomas[22] found amongst them no elevation of blood-pressure and rarely any evidence of renal disorder. They do not show any harm attributable to their high protein intake.

Observations made on diabetics suggest that the early onset of atheroma to which diabetics were prone was due to the high fat régime to which they used to be condemned.[23] There has been a diminution in this liability to arterial degeneration since insulin has enabled diabetics to take carbohydrate in normal proportions instead of looking to fats for their calories. Unregulated dosage of infants with vitamin D may involve some risk of hypervitaminosis, but so far this condition has only been recognized in laboratory animals to whom massive doses have been administered.[24] The effects are hypercalcification of the epiphysis and calcification of the circulatory systems of the stomach and kidneys. The products of fat metabolism seem capable of doing more damage to our arteries than the products of protein metabolism.

*Diet and Renal Disease.*

The observations of the Eskimos show that their high meat diet does not lead to any undue frequency of renal disease. Stefansson,[25] the arctic explorer, has lived in Eskimo fashion on an all-flesh diet for many months without ill-effects. Jackson and Riggs,[26]

by feeding rats on a diet rich in protein over long periods did not cause them to develop chronic nephritis. In the treatment of acute nephritis limitation of the protein intake is imperative because of the dangers of nitrogen retention and uræmia. Even cow's milk contains too much protein (4 per cent.) to form the exclusive diet in acute nephritis, although it was formerly advised. Nitrogenous food may be entirely cut off during the first few days, at any rate, and a carbohydrate diet should be prescribed. Chloride retention occurs, and for this reason it is customary to forbid the use of common salt; but the view that chloride retention plays a chief part in the production of œdema is probably not the whole truth. For in dealing with chronic parenchymatous nephritis protein depletion governs in some degree the amount of œdema, just as it does in famine dropsy.[27] This was turned to account in the Epstein high protein diet,[28] which is designed to raise the low protein content of the blood whilst it limits the fat intake in order to avoid lipæmia. This diet is applicable to cases who show a low blood urea content and whose urine is capable of a urea concentration approximating 2 per cent.

*Diet and Hepatic Disease.*

Next to nothing is known of the effect of diet on the liver and its functions apart from the admitted damage which alcohol can inflict. I have already mentioned the protective value of carbohydrates against toxæmias of pregnancy and certain metallic poisons. A good glycogen store in the liver is also a protection against the delayed effects of chloroform, and it certainly serves as a reserve source of energy which is drawn upon during exhausting muscular exertion.

*Diet and Endocrine Functions.*

The most important bearing of diet upon the endocrine glands is the value of iodine in preventing endemic goitre or cretinism. Simple goitre depends upon the absence of iodine in the diet. In Switzerland 1 to 5 milligrammes taken daily in table salt protects the population. Cases of Graves's disease, as a rule, show temporary improvement when iodine is administered, and this amelioration is often made use of in the preparation of patients for the operation of thyroidectomy.

Feeding with raw thyroid gland and extracts of thyroid is now an accepted mode of curing thyroid deficiency, but does not come within the scope of my address. Neither am I concerned with the cure of tetany by means of the administration of parathyroid extract and calcium, since these are not illustrations of the prevention of disease by dietetic means, but rather of cure of already existing disease.

*Diet and Gout.*

It might be feared that this section of my address will occupy the remainder of the time at our disposal, but this is far from being the case. Whilst it is universally supposed that indiscretion in diet is the prime cause of gout, no one as yet has been able to define a gouty indiscretion nor tell us how indiscreet we have to be to incur the penalty of gout. That gout is related in some way to the disposal of purins and their end-products is certain, but the ingestion of exogenous purins does not provoke gout in the majority of individuals, nor does their avoidance by any means protect the gouty from further gouty deposits either in an acute or chronic form. Large meals and deep potations may exacerbate the manifestations of gout, but the prime causes of the disease probably lie amongst the so-called "inborn errors of metabolism." I do not think there is any scientific evidence showing that by modification of diet gout can be produced in every individual in the same sense that rickets or scurvy can be. What I mean is that it is not proved that by any given food-stuff or dietary the error of metabolism causing gout can be originated. Hale White,[29] in 1906, said he thought gout was very little dependent on animal food, for the consumption of meat had during the past fifty years increased from 3 lbs. a head per year to 50 lbs., yet gout had steadily diminished. Stendel and Ellinghaus[30] say that the German war bread produced intestinal fermentation which destroyed the purins of the diet and gout virtually disappeared among the population.

*Diet and Diabetes.*

Diet is largely to blame for diabetes. The aphorism that every human being is a potential diabetic appears to stand the test of time and is founded upon observations in India. Amongst the merchant classes of India diabetes has been said to indicate worldly success. In other words, over-indulgence in carbohydrates leads to hyperglycæmia and no pancreas can long withstand hyperglycæmia. Degeneration of the islets of Langerhans results sooner or later. Individual variations, of course, exist in the tolerance for ingested carbohydrate, but a point is reached in almost everyone where the blood-sugar will be permanently elevated above normal by constant excess of carbohydrate in the food. Not every case of diabetes depends upon this dietetic excess ; inflammatory changes may damage the islets, so too may vascular degeneration. These may be unavoidable accidents, but it is unquestionable that there remain a large number of diabetic patients whose disease has been produced by carbohydrate excess and might have been prevented by the simplest regulation of their food.

Even in the diabetic patient some of the most serious accidents and complications can be produced and prevented through the food. Coma, which was formerly the main danger in diabetes has almost disappeared from the death statistics since we learned to balance the fat intake with enough carbohydrate to ensure complete combustion of the fats. Atheroma and arterio-sclerosis used to occur prematurely in nearly all diabetics ; now that insulin has enabled them to take adequate carbohydrate and avoid excessive fat dietaries they can look forward to a hale old age unthreatened by premature arterial decay. This is not determined by mere injection of insulin, it is the direct result of carbohydrate feeding as contrasted with fat.

*Diet and Rheumatism.*

The approach to this topic demands the most delicate of treads. Not so very long ago chronic arthritis of any description was attributed to a faulty diet, and lavish promises of cure were made to the victims. That careful study of chronic rheumatic diseases written by our Bath colleagues Thomson and Gordon [31] is free from any tendency to hold food responsible for chronic arthritis. They will commit themselves no farther than to say : " It is suggested that in those prone to fibrositis there may be some functional inferiority of the lower digestive tract, which allows either the growth of an abnormal organism or the passage of toxins derived from improperly digested food." You will notice that this is advanced as no more than a suggestion. In the *British Medical Journal* [32] of 25th October, 1930, there is a statement bearing on these suggestions which sums up our knowledge or lack of it: "Following on the idea that stasis encouraged absorption of poisons from the bowel contents, search was made for these poisons, but so far no such poison has been obtained from either bacterial or chemical products recovered from the intestinal contents."

Some patients afflicted with chronic rheumatism in one or other form find that particular modifications of dietary secure to themselves individual relief and comfort, but no general principles have been evolved from these experiences, and no amount of research has determined the explanation of the relief, and even in some cases the definite retrogression of the disease which has been noted.

A dietetic study of two native tribes in East Africa has been described by Orr.[33] One tribe was almost entirely vegetarian, their diet was found to be deficient in calcium, and suspected of being deficient also in protein and certain vitamins; the other was largely carnivorous, their food consisted mainly of meat, milk and blood, and was rich in protein, calcium and certain of the vitamins believed to be lacking from the vegetarian diet. Rheumatoid arthritis is described as common in the carnivorous tribe and rare in the vegetarian, the percentage of this disease out of the total cases of sickness being 35 per cent. in the meat eaters and only 2 per cent. amongst the vegetarians. The observations are suggestive, but the composition of the diets is not known with precision, and the causal connection between the presence or absence of meat-protein and rheumatoid arthritis is only conjecture.

One might with an almost equally justifiable conjecture attribute the absence of acute rheumatism amongst the natives of India to some dietetic factor, because their diet is so completely different from that of the races where acute rheumatism is common.

*Diet and Infection.*

Graves,[34] one of the wisest of physicians, wrote of the Irish famine of 1847: "A vast amount of mischief was produced by the attempt to connect fever epidemics with a deficiency of food." He deplored that "the text put forth so authoritatively, if there be no famine there will be no fever, prevented proper attention from being paid to the real causes which produce and promote the spread of epidemic diseases." Graves would admit no more than that "want of a sufficiency, or food of an unwholesome or improper character, predisposes the human frame to disease by its debilitating effects on the system. . . . I cannot admit that either cause is sufficient to generate an epidemic." He repeats and associates himself with a staggering indictment: "Pestilence has followed the footsteps of benevolence," but explains that charity attracted crowds, and charity, rather than turn away the sick and destitute, overcrowded the hospitals and workhouses, and fevers, relapsing, typhus or small-pox, it might be, were disseminated through crowding and not by lack of nourishment.

Even now, seventy years after those words were written, it is hard to improve them: "Want of food or unwholesome food or food of an improper character predisposes the human frame to disease by its debilitating effects on the system."

Funk,[35] reviewing all the claims advanced to support the thesis that vitamin deficiencies are responsible for impaired resistance to infections, admits that A appears to be more directly related to resistance than any other food factor, and concludes that it is not possible to prove as yet a definite relation between dietetic deficiency and inflammatory processes of the nasal sinuses, middle ear disease, pneumonia and ulcerative conditions of the mouth and eyes.

Caffey [36] observes that the failure of the healthier and the better nourished child to escape the acute infectious diseases, such as measles or influenza or even cerebro-spinal meningitis, is an obvious contradiction to the principle that good nutrition confers immunity to infection.

A careful analysis of 6,984 children under five years old during a period of twelve years made by Barenberg [37] and others showed no evidence that nutrition influenced the morbidity or mortality from pneumonia. A second finding (I hardly dare quote it lest it should be misinterpreted) was that in 114 cases of rickets the incidence of pneumonia was only one quarter that of 102 non-rachitic children of the same age group, thirteen to eighteen months. During the war it was amazing to see how the healthy, well-fed men of magnificent physique from the sheep runs of Australia and the farmlands of Western America succumbed to respiratory diseases which the denizens of our worst slums resisted best. Some other factors than nutrition were accountable for this immunity of the slum-dweller. Even the association of malnutrition with the development of tuberculosis is open to question because of the difficulty of making sure that the so-called predisposing malnutrition was not due to the tuberculous infection in an early stage.

Schultze and Zilva [38] surveyed this problem in the *Journal of Hygiene* in 1927, and concluded that there was insufficient evidence to prove that diets deficient in fat soluble vitamins rendered laboratory animals more susceptible to tuberculous inoculation, and that feeding guinea-pigs so inoculated with cod liver oil did not prolong their lives, improve their weight curves, or curtail the extent of the disease.

Gerson's diet, in which chlorides are withdrawn and various other mineral salts are added, has been advocated as a protection against the progress and activity of tuberculosis. Lambotte [39] published in the *Liége Médical* last January an extensive summary of various authors' experiences with this diet. The results resemble those of all other remedies for tuberculosis. The authors report the most contradictory findings except in reference to lung disease, where the efficacy of Gerson "transmineralization" plan is held definitely to have failed. It is worth further trial, says Lambotte, in tuberculous lesions and, above all, in lupus.

In scurvy there is an admitted predisposing factor which permits of infection of the gums by organisms which set up gingivitis and pyorrhœa. My personal experience in the condition known as trench mouth convinced me that in most cases there was not only an infection by Vincent's spirillum and *bacillus fusiformis*, but that there was an associated mild degree of scurvy, so that prevention, quite as much as cure, depended on furnishing the men with adequate sources of vitamin C. Mild grades of scurvy are fairly common amongst the senile cases at our Poor Law hospitals, and it is usually pyorrhœa with gingivitis which attracts attention to them. Höjer, quoting Hess,[40] says: "One of the most striking and important symptoms of scurvy is the marked susceptibility to infections, furunculosis, nasal diphtheria, grippe, bronchitis and pneumonia."

McCarrison and others have demonstrated that the intestinal mucous membrane of animals fed with a B deficiency is especially prone to bacterial invasion. Cramer[41] regards A as essential to the normal development of the mucous membrane of the bowel. Rats fed on diets partly deficient in A showed atrophic and destructive changes in the intestinal mucous membrane, leading, as he thinks, to invasion by micro-organisms. On the other hand, he thinks motor disturbances of the bowel are associated with the anti-neuritic $B_1$ vitamin.

Haas[42] seeks to connect cœliac disease in children with a partial lack of $B_1$, and believes that cœliac disease may so interfere with assimilation of $B_1$ that beri-beri develops. Which is cause and which effect may be uncertain, but certainly beri-beri is usually associated with changes in the alimentary tract. But it is unjustifiable to go to the extremes of attributing all bowel irregularities to lack of $B_1$, and it is absurd to suppose that we are dependent upon eating wholemeal flour or bread for our sole supply of this vitamin.

The table on page 281 presents a summary of the chief facts ascertained about vitamins.

*Diet and Mental Disease.*

The mental arrest of cretinism may be classed among the disorders influenced by diet food in so far as some cases are attributable to a lack of iodine in the diet. Mental disorder culminating in dementia is the rule in pellagra accompanied by cord and nerve changes. Pellagra is now attributed to a lack of a vitamin designated $B_2$, PP or G.

A most interesting observation has been made at the Besford Court Catholic Mental Welfare Hospital[43] for Children. An epidemic of boils in this institution led the authorities to increase the fat ration with the object of supplying vitamin A. The fat was furnished in the form of beef dripping. Not only was the

### VITAMINS.

| Designation .. | A. | $B_1$ or F. | $B_2$ or PP or G. | C. | D. | E. |
|---|---|---|---|---|---|---|
| Solubility .. | Fat. | Water. | Water. | Water. | Fat. | Fat. |
| Effects :<br>Prevents .. | Xerophthalmia.<br>Infection. | Beri-beri.<br>Neuritis.<br>Atrophy of lymphoid tissue. | Pellagra. | Scurvy.<br>Dental decay. | Rickets.<br>Dental decay. | Sterility. |
| Promotes .. | Growth. | Growth. | | Growth. | Development of :<br>(*a*) Teeth and their enamel.<br>(*b*) Bone. | Fertility. |
| Sources .. .. | Cod liver oil.<br>Cream.<br>Butter.<br>Milk.<br>Beef-fat.<br>Liver.<br>Egg-yolk.<br>Green vegetables.<br>Carrots.<br>Tomatoes.<br>Oranges.<br>Destroyed by ultra-violet radiation. | Yeast.<br>Germ of cereals.<br>Peas, beans, lentils.<br>Nuts.<br>Eggs.<br>Fresh meat.<br>Liver. | Yeast.<br>Germ of cereals.<br>And a little in peas, beans and lentils.<br>Eggs.<br>Fresh meat.<br>Fish.<br>Eggs.<br>Cheese.<br>Wholemeal flour.<br>Tomatoes. | Fresh vegetables.<br>Fresh fruit.<br>Milk.<br>Fresh meat.<br>Germinated cereals and pulses.<br>Lemon-juice.<br>Tinned tomatoes, but *not* Lime-juice. | Cod liver oil.<br>Fish-roe.<br>Cream.<br>Cheese.<br>Butter.<br>Egg-yolk.<br>Liver.<br>" Viosterol " (or irradiated ergosterol). | In practically all these foods. |

physical condition of the boys greatly benefited, but, in the words of the report, "two or three weeks after the new dietary had been in use conduct disorders vanished; in place of a general feeling of discomfort a spirit of cheerfulness appeared, adverse reports upon boys became rare, and boys whose reactions . . . had been lackadaisical or negative woke up as it were and began to take a real interest in their work."

*Diet and Nervous Diseases.*

The neuritis which occurs in beri-beri is preventable by vitamin $B_1$. In diabetes we see a peripheral neuritis which improves when the sugar content of the blood is restored to normal limits. Pernicious anæmia is often accompanied by sub-acute combined degeneration of the spinal cord. Liver and ventriculin therapy are too recently introduced to make it possible to say whether by this means the cord changes can be prevented. Certainly when the sclerosis has progressed sufficiently to be manifest liver therapy can do little good.

Epilepsy, which I have mentioned previously as occasionally a food allergy, has been found in some cases remarkably amenable to treatment by the ketogenic diet, that is a diet in which the fats are increased and carbohydrates are diminished to a point which will produce ketosis. Not only is the incidence of fits and attacks of *petit mal* lessened, but there is a marked improvement in behaviour, so that the demeanour of a hitherto incorrigible boy may change completely.

### CONCLUSION.

Once upon a time it was pretended that men, women and children could be made healthy if doctors were given plenary powers to issue hard and fast orders in the matter of dietaries, but with increasing scientific knowledge our pretensions in this direction must be less cocksure. Our advice must be for the most part limited to warnings of the evil effects of omitting essential foods. We must begin to doubt our ability to guide even the youngest infants aright. An instinctive choice of the superior food above the inferior exists in rats and mice. When the appetite is given full control of what shall be eaten it is surprising to note how pigs naturally select the specific feeds which swine herdsmen have long since approved of as best, and, what is equally surprising, the pigs show a marked avoidance of those feeds usually considered ill-adapted to swine.

We can learn from babes and sucklings the same lesson. Clara Davis[44] published in 1928 a paper entitled "The self-selection of diet by newly-weaned children." Three infants newly-weaned were allowed to choose their own foods in such quantities as they pleased from a fairly wide range of commonly used natural food materials, unmixed, unseasoned and unaltered except in the case of some by cooking in the simplest manner. The food was not offered directly or by suggestion. But when, and only when, the infant reached for or pointed to a dish the nurse might take up a spoonful and if he opened his mouth for it put it in without comment or any refusal or correction of his manners. The result was that after the first few meals foods wanted were promptly recognized and chosen without hesitation whatever their position on the tray. The children soon learned to help themselves, they were not guided by bright colours. They showed themselves omnivorous with a liking for most foods on the list, but rarely ate more than three solids at one meal. They showed decided preferences, but in spite of this it proved impossible to predict what would be eaten at a given meal, *e.g.* an infant might eat from one to seven eggs or none and from one to four bananas. Even the daily consumption of milk was unpredictable, varying from 11 to 48 oz. A tendency was observed for them to eat certain food in waves. They would indulge in meat jags, egg jags and cereal jags, but they did not show disgust from surfeit, as they would continue these foods at the former level. Judged from every point of view, the diet selected by these infants was optimal, equal at least to the best results obtained by commonly prescribed diets in growth, weight, bone development, musculature, general vigour and appearance of health and well being. The experiment lasted in the case of two infants for a period of six months and the third a year. It demonstrates that the transition from the suckling diet to one embracing the natural foods of adult life need not be made such a gradual process as is commonly assumed.

When I chose Food as the subject of this lecture I felt grave misgivings as to its appropriateness. I doubted whether it would have been approved by Dr. Long Fox; but I took heart of grace from an incident in his life recorded by Munro Smith in his *History of the Bristol Royal Infirmary.*[45] Dr. Long Fox felt that a good medical library and a large and convenient room for professional meetings was needed in the University College. On 2nd June, 1888, he gave a dinner to a number of medical men at the Queen's Hotel, Clifton, and advocated the establishment of such a library. So successful was he, and so shrewdly did he appreciate what one might call the biological value of such a dinner, that no less a sum than £1,200 was promised by those present at the dinner. The room built with those subscriptions is now the Senate Room of the University.

REFERENCES.

[1] Copeman and Greenwood, *Diet and Cancer*, Ministry of Health Report on Public Health and Medical Subjects, No. 36, 1926.

[2] Lewis, *The Blood Vessels of the Human Skin and their Responses*, London, 1927.
Harris, *Heart*, 1927, xiv. 161.
Lewis and Grant, *Heart*, 1924, xi. 209.

[3] Naumann, *Deutsche klin. Wchnschr.*, 1929, lv. 2,143.

[4] Bradfield, *Indian M. Gaz.*, 1930, lxv. 426.

[5] Murray, *Report of Imperial Cancer Research Fund*, 1927–28.

[6] Rohmann, *Klin. therap. Wchnschr.*, 1902, ix. 1,306; *Ueber künstliche Ernährung und Vitamine*, 1916; quoted by Lusk, *The Science of Nutrition*, Philadelphia, 4th Ed., 1928, 487.

[7] Pekelharing, *Nederlandsch Tijdschrift voor Geneeskunde*, 1905, 111; quoted by Lusk, *op. cit.*, 487.

[8] Hopkins, *Analyst*, 1906, xxxi. 391; quoted by Lusk, *op. cit.*, 488.

[9] Lusk, *op. cit.*, 516.
Willcock and Hopkins, *J. Physiol*, 1906–7, xxxv. 88.

[10] Hess, *Rickets, including Osteomalacia and Tetany*, Philadelphia, 1929.

[11] Dalyell and Chick, *Lancet*, 1921, ii. 842.
Stapleton, *Lancet*, 1925, i. 1,119.

[12] Mellanby and Pattison, *Brit. M. J.*, 1928, ii. 1,079.

[13] Caffey, *Progressive Medicine*, March, 1930, 252.

[14] Hoobler, *J.A.M.A.*, 1928, xci. 307.
Caffey, *Progressive Medicine*, March, 1930, 250.

[15] Hoefler and Hardy, *J.A.M.A.*, 1929, xcii. 615.

[16] Riegel, *Nothnagel's Encyclopedia*, "Diseases of the Stomach," 179.

[17] Thomas; quoted by Lusk, *op. cit.*, 478.

[18] Rubner, *Arch. f. Physiol.*, 1918, 135.
Lusk, *op. cit.*, many references.

[19] Lusk, *op. cit.*, 58.

[20] Alvarez, *J.A.M.A.*, 1919, lxxii. 8; quoted by Wright, *Applied Physiology*, London, 1928, 411.

[21] Brem, Zeiler and Hammack, *Am. J. M. Sc.*, 1928, clxxv. 96.

[22] Thomas, *J.A.M.A.*, 1927, lxxxviii. 1,559; quoted by Lusk, *op. cit.*, *ibid.*, 20th September, 1930, Ed., 458.

[23] Joslin, *Treatment of Diabetes Mellitus*, London, 1928, 685.

[24] Hess and Lewis, *J.A.M.A.*, 1928, xci. 783.

[25] Stefansson, *The Friendly Arctic*, London, 1921; quoted by Lusk, *op. cit.*, 458.

[26] Jackson and Riggs, *J. Biol. Chem.*, 1926, lxvii. 101; quoted by Lusk, *op. cit.*, 692.

[27] Nixon, *Official History of the Great War: Medical Services—Diseases of the War*, i. 450.
Maver, *J.A.M.A.*, 1920, lxxiv. 934.

[28] Box, *Brit. M. J.*, 1920, i. 356.
Epstein, *Am. J. M. Sc.*, 1917, cliv. 636.

[29] Hale White, *Lancet*, 1906, ii. 1779.

[30] Stendel and Ellinghaus, *Ztschr. f. physiol. Chem.*, 1923, cxxvii. 291; quoted by Lusk, *op. cit.*, 736.

[31] Thomson and Gordon, *Chronic Rheumatic Diseases*, London, 1926.

[32] *Brit. M. J.*, 1930, ii. 695.

[33] Orr, *Report on Investigations on Mineral Deficiencies in Pastures of Kenya Colony and Effects on Grazing Animals*, H.M.S.O., 1930; quoted in *Transactions Highland and Agricultural Society of Scotland*, 1930, xlii. 28.

[34] Graves, *Clinical Medicine*, i., 106 (New Syd. Soc.).

[35] Funk, *Progressive Medicine*, June, 1929, 266.
Green and Mellanby, *Brit. M. J.*, 1928, ii. 691.
Sherman and Burtis, *Proc. Soc. of Exper. Biol. and Med.*, 1928, xxv. 649.

[36] Caffey, *Progressive Medicine*, March, 1930, 263.

[37] Barenberg, Green and Abrahamson, *J.A.M.A.*, 1929, xcii. 441.

[38] Schultze and Zilva, *J. Hyg.*, 1927, xxvi. 204.

[39] Lambotte, *Liége Méd.*, 1930, xxiii. 1.

[40] Höjer, *Acta Pædiat.*, 1924, iii. (Supp.), 119.

[41] Cramer, *Lancet*, 1924, i. 633.
Fletcher, *J. Lab. and Clin. Med.* 1930, xv. 1,140.

[42] Haas, *Arch. Pediat.*, 1929, xlvi. 467.

[43] *Tenth Annual Report of Besford Court Catholic Mental Welfare Hospital for Children*, 1927–8, 82.

[44] Davis, *Am. J. Dis. Child.*, 1928, xxxvi, 651.
Wright and Geddes, *Canad. M. A. J.*, 1930, xxiii. 537.

[45] Munro Smith, *History of the Bristol Royal Infirmary*, Bristol, 1917, 478.

